# A mobile-based system for maize plant leaf disease detection and classification using deep learning

**DOI:** 10.3389/fpls.2023.1079366

**Published:** 2023-05-15

**Authors:** Faiza Khan, Noureen Zafar, Muhammad Naveed Tahir, Muhammad Aqib, Hamna Waheed, Zainab Haroon

**Affiliations:** ^1^ University Institute of Information Technology, Pir Meh Ali Shah (PMAS)-Arid Agriculture University, Rawalpindi, Pakistan; ^2^ Data Driven Smart Decision Platform for Increased Agriculture Productivity, Pir Meh Ali Shah (PMAS)-Arid Agriculture University, Rawalpindi, Pakistan; ^3^ Department of Agronomy, Pir Meh Ali Shah (PMAS)-Arid Agriculture University, Rawalpindi, Pakistan; ^4^ National Center of Industrial Biotechnology, Pir Meh Ali Shah (PMAS)-Arid Agriculture University, Rawalpindi, Pakistan; ^5^ Department of Land and Water Conservation Engineering, Faculty of Agricultural Engineering and Technology, Pir Meh Ali Shah (PMAS)-Arid Agriculture University, Rawalpindi, Pakistan

**Keywords:** deep learning, object detection, YOLO, transfer learning, disease classification

## Abstract

Artificial Intelligence has been used for many applications such as medical, communication, object detection, and object tracking. Maize crop, which is the major crop in the world, is affected by several types of diseases which lower its yield and affect the quality. This paper focuses on this issue and provides an application for the detection and classification of diseases in maize crop using deep learning models. In addition to this, the developed application also returns the segmented images of affected leaves and thus enables us to track the disease spots on each leaf. For this purpose, a dataset of three maize crop diseases named Blight, Sugarcane Mosaic virus, and Leaf Spot is collected from the University Research Farm Koont, PMAS-AAUR at different growth stages on contrasting weather conditions. This data was used for training different prediction models including YOLOv3-tiny, YOLOv4, YOLOv5s, YOLOv7s, and YOLOv8n and the reported prediction accuracy was 69.40%, 97.50%, 88.23%, 93.30%, and 99.04% respectively. Results demonstrate that the prediction accuracy of the YOLOv8n model is higher than the other applied models. This model has shown excellent results while localizing the affected area of the leaf accurately with a higher confidence score. YOLOv8n is the latest model used for the detection of diseases as compared to the other approaches in the available literature. Also, worked on sugarcane mosaic virus using deep learning models has also been reported for the first time. Further, the models with high accuracy have been embedded in a mobile application to provide a real-time disease detection facility for end users within a few seconds.

## Graphical Abstract

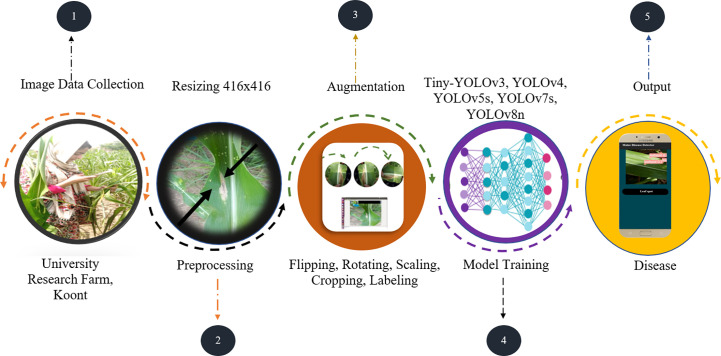

## 1 Introduction

Agriculture is considered the backbone of Pakistan’s economy, which is reliant on key crops such as wheat, rice, and maize (Shar et al., 2021). It is considered the primary source of income for the large population in Pakistan. One of the major causes of low yield is diseases, which reduce the quality, quantity, and nutritional value of fruits, vegetables, cereals, and legumes (Saleem et al., 2019; Turkoglu et al., 2019). Nearly 32% of the losses are observed due to diseases in cereal crops (Tudi et al., 2021). Maize is very essential crop all around the world, especially in Pakistan. It is used for different purposes i.e., poultry, cattle feed, food, beverages, etc. It contributes 19% (Abdullah et al., 2021) to the Gross Domestic Product (GDP) but unfortunately, it is prone to various diseases which results in low production. Some major maize plant diseases are Blight (Sun et al., 2020), Sugarcane Mosaic virus (Liu and Wang, 2021), and Leaf Spot (Krawczyk et al., 2021). The diseases look the same in their emerging stages, hence difficult to differentiate from the human eye and are also time-consuming. Therefore, Artificial Intelligence (AI) technologies have become one of the current research hotspots based on the above problem.

AI and deep learning-based methods are progressively being utilized in agricultural research, because of their ability to automatically learn the deep features from the image dataset, also their accuracy and speed levels are higher than the traditional algorithms (Chen et al., 2022). The most popular architecture of deep learning is Convolutional Neural Network (CNN). Specifically, it was intended to work with images, video recognition, medical image analysis, object detection, flow prediction (Miglani and Kumar, 2019), traffic control (Jindal and Bedi, 2018), recommendation systems for healthcare (Shaikh et al., 2022), anomaly detection (Garg et al., 2019), recognition of diseases (Astani et al., 2022), weed detection (Khan et al., 2022), soil monitoring (Chen S. et al., 2022), and pest identification (Cheng et al., 2021; Huang et al., 2022), etc.

Nowadays, deep learning-based approaches, including Single Shot Detector (SSD) (Sinan, 2020), You Only Look Once (YOLO) (Ponnusamy et al., 2020; Ganesan and Chinnappan, 2022) models, etc. are being used for leaf disease classification and recognition. (Cheng et al., 2022) have proposed the improved YOLOv4 which is based on lightweight CNN and was developed for weed detection and seedling of maize crop. They have trained their model on the 1800 images. First, they decrease the number of parameters and increase the speed of feature extraction, also MobileNetv3 is used for building the lightweight feature extraction backbone network to replace the CSPDarkNet53 network in +YOLOv4. Secondly, they applied the transfer learning technique which is used for increasing the training speed. The model gives the Mean Average Precision (*mAP*) of 89.98%, the detection speed is 69.76 FPS, and the number of parameters was 8.17x106. However, the accuracy of the model could be improved also the dataset should be larger. According to Shill and Rahman, 2021, the detection results for plant disease by using the YOLOv3 and YOLOv4 were obtained as 53%, 52% *mAP*, and 55%, 56% F1-score, respectively. The overall *mAP* of YOLOv4 is better than that of the YOLOv3 model, however, the dataset was collected in the lab setting from PlantDoc repository for model training.

Roy and Bhaduri (2021) have developed a real-time object detection framework that is based on an improved YOLOv4 algorithm, and they applied this algorithm to detect apple plant diseases. They acquired the dataset from Kaggle which consists of two diseases of apple plant total of 600 images of each class. The proposed model was modified to enhance accuracy and then it was verified in the orchard with complex background. The algorithm obtained a *mAP* value of 91.2% with 56.9 FPS and F1-score value is 95.9%. The modified model compared with the original YOLOv4 model showed that the developed model gives a 9.05% increase in *mAP* and 7.6% in F1-score. Gokulnath and Usha Devi (2020) have developed the BOOSTED-DEPICT model that can do clustering of maize images and many other techniques called k-means clustering, deeply embedded clustering, and regularized deep clustering and has achieved the accuracies of 97.73% and 91.25% on Plant Village (PV) and PDD dataset, respectively. Nayar et al. (2022) have used the YOLOv7 model for the detection of different plant diseases that were collected from the Plant Village dataset and achieved 65% *mAP*, the results reported in their study don’t meet the real-time detection system. Chen et al. (2022) have proposed an improved YOLOv5 model to detect the rubber tree disease (powdery mildew) with 2375 images and the model obtained 70% *mAP*. The improved version of YOLOv5 gained 5.4% higher results than the original YOLOv5.

While considering the above discussion, to the best of our knowledge there are fewer attempts in the real-time classification, detection, segmentation, and tracking system of maize crop diseases, also the sugarcane mosaic virus disease is neglected in the literature. The real-field dataset is not publicly available for training and testing of the model, also image dataset used by different authors in the literature is limited to a lab environment which is not suitable for real-time scenarios. Therefore, we have collected the dataset from the real field with a heterogenous background, on contrasting weather, and different lighting conditions. Moreover, we have developed smartphone application for the quick assessment of maize crop. The primary contributions of this paper are as follows:

- A real disease data repository is built containing images of maize crop, collected from the university research farm, located in the Potohar region of Punjab province, Pakistan.
- This repository contains images of three distinct types of diseases acquired in different weather conditions and timestamp values. It also includes the data of sugarcane mosaic virus disease which is not reported in any deep learning-based detection work in the past.
- The state-of-the-art YOLOv8n model for maize plant disease detection and classification is used.
- The best-trained model is integrated with the user-friendly smartphone application for the real-time detection of maize disease to facilitate the end user.

The rest of the paper is organized as follows. The background is presented in section 2. Materials and methods are discussed in section 3. Section 4 elaborates the proposed system’s results and discussion, and section 5 concluded the discussion.

## 2 Background

YOLO is a SOTA algorithm that is used for real-time object detection, classification, segmentation, and tracking (Jiang et al., 2021). It is the most popular algorithm for speed and accuracy aspects. In our research, maize foliar diseases are trained on YOLOv3-tiny (Malta et al., 2021), YOLOv4, YOLOv5s, YOLOv7s, and YOLOv8n because they are fast, accurate, and real-time object detection algorithms.

### 2.1 YOLOv3-tiny

**Figure 1:**
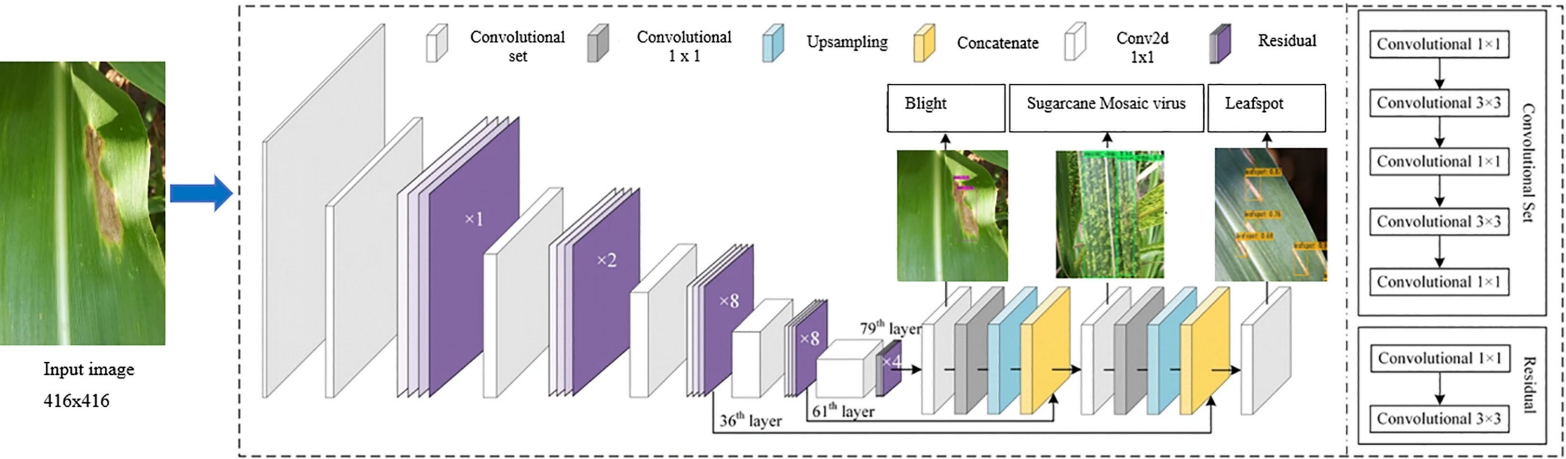
Architecture diagram of YOLOv3-tiny.

The YOLOv3-tiny model is a simplified version of YOLOv3. It has a smaller number of CNN layers and utilizes less memory. In YOLOv3, Darknet-53 is used as a deeper architecture for feature extraction. It means 53 CNN layers have been used, and each CNN layer is followed by the Leaky Rectified Linear Unit (Relu) activation function Equation (1), where x represents the input (Agarwal et al., 2021). It is based on the ReLU activation function but with a small slope or negative values rather than a flat slope. Also, it gives the benefit of fast training. The hyperparameters used for training the YOLOv3-tiny are given in **Table 1**. The architecture diagram of YOLOv3-tiny is illustrated in **Figure 1**.

**Table 1:** Hyperparameters configuration for YOLOv3-tiny, YOLOv4, YOLOv5s, YOLOv7s, YOLOv8n.

Model	Hyperparameters	Value
YOLOv3-tiny	alpha batch size Subdivisions Activation function	0.001 64 16 Leaky-ReLU
YOLOv4	max batches alpha batch size Subdivisions Activation function	6000 0.001 64 16 Mish
YOLOv5s	Optimizer Momentum alpha batch size Activation function Weight decay	Stochastic Gradient Descent 0.937 0.01 64 Mish 0.005
YOLOv7s	Alpha Momentum Optimizer Weight decay Activation function Batch size Warmup epochs Warmup bias lr (learning rate)	0.01 0.937 Stochastic Gradient Descent 0.0005 leaky ReLU 64 3.0 0.1
YOLOv8n	alpha momentum weight decay warmup epochs warmup momentum Warmup bias lr Optimizer Batch size Activation function	0.01 0.937 0.0005 3.0 0.8 0.1 Adam 64 SiLU


(1) $$Leaky-ReLU\left(x\right)\;=\;max\left(ax,x\right)\;=\;\{\begin{array}{c} ax,if\;x>0\\ x,if\;x\geq 0 \end{array}$$

Where *a* ϵ [ 0,1]

### 2.2 YOLOv4 model

**Figure 2:**
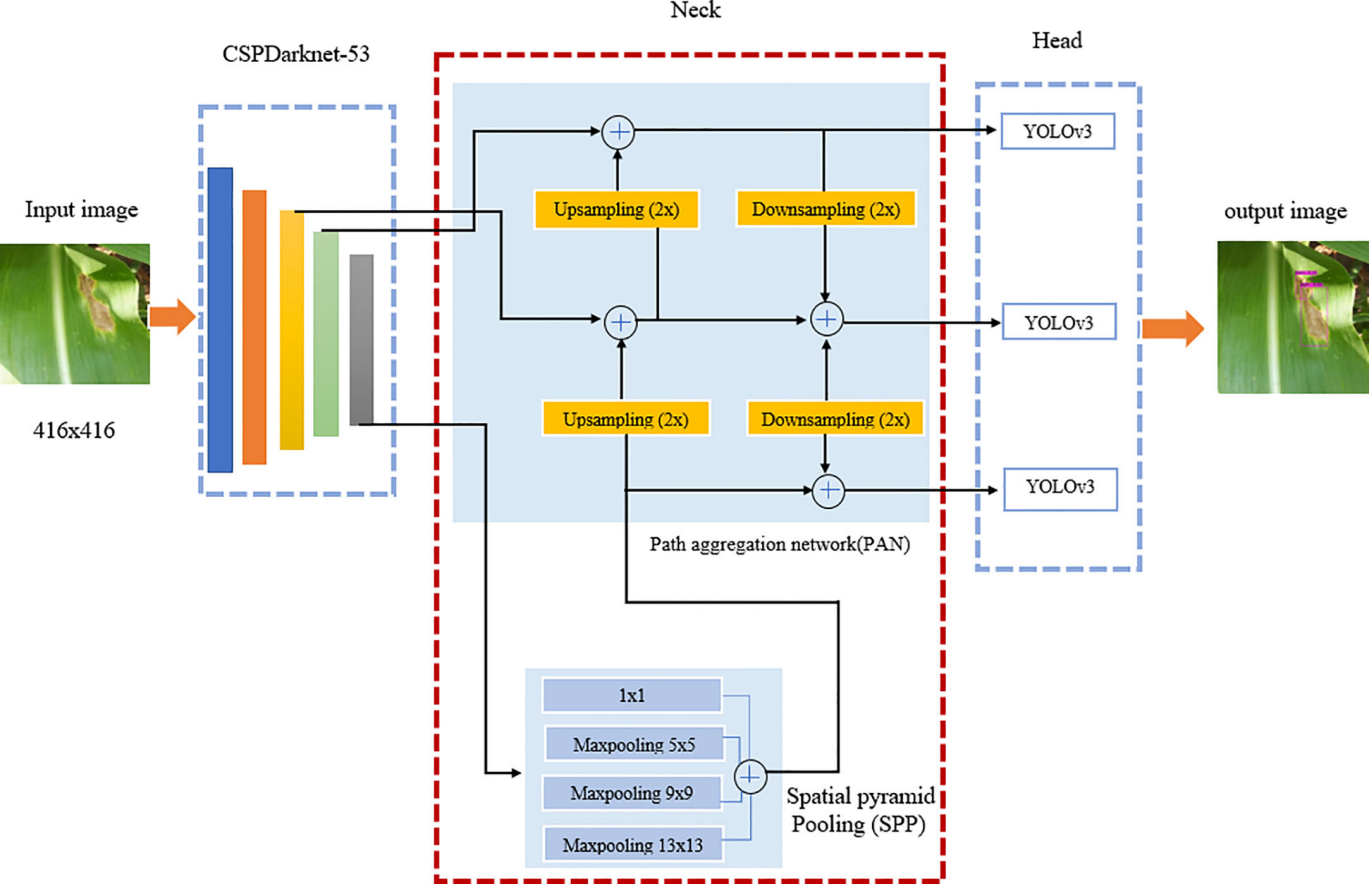
Architecture diagram of YOLOv4.


YOLOv4 is the advancement of the YOLOv3 model in the *mAP* by as much as 10% and frames per second (FPS) by 12%. It is a one-stage object detection model composed of three parts; the first is the backbone, the second part is the neck, and the last part is the head. The backbone is pre-trained CNN on Center and Scale Prediction (CSPDarknet53). The CSPDarket53 means that the model consists of 53 CNN layers and this stage is responsible for extracting the features and computing feature maps from the input images. The neck section is responsible for concatenating the backbone with the head. The neck section is composed of a spatial pyramid pooling (SPP) and a Path Aggregation Network (PAN). The neck collects the feature maps from the backbone and feeds them into the head as input. In the last section, the head is responsible for processing the aggregated features and predicting the bounding box, prediction score classifies into the relevant class. In our study, the image size is fixed to 416×416, for the training and testing process. The architecture of the YOLOv4 model is shown in **Figure 2**. The hyperparameters used in YOLOv4 for training purposes are shown in **Table 1**. The activation function used here was Mish as shown in Equation (2). Among other activation functions, Mish is the best choice because it is smooth, and non-monotonic (Mathayo and Kang, 2022). It has various properties which made it popular from Swish and Rectified Linear Unit (ReLU), like unbounded above and bounded below improves its performance and it is low cost.

(2)  f(x) = xtanh(softplus(x))

Where *softplus* (*x*) = In(1+*e^x^*)

### 2.3 YOLOv5s model

**Figure 3:**
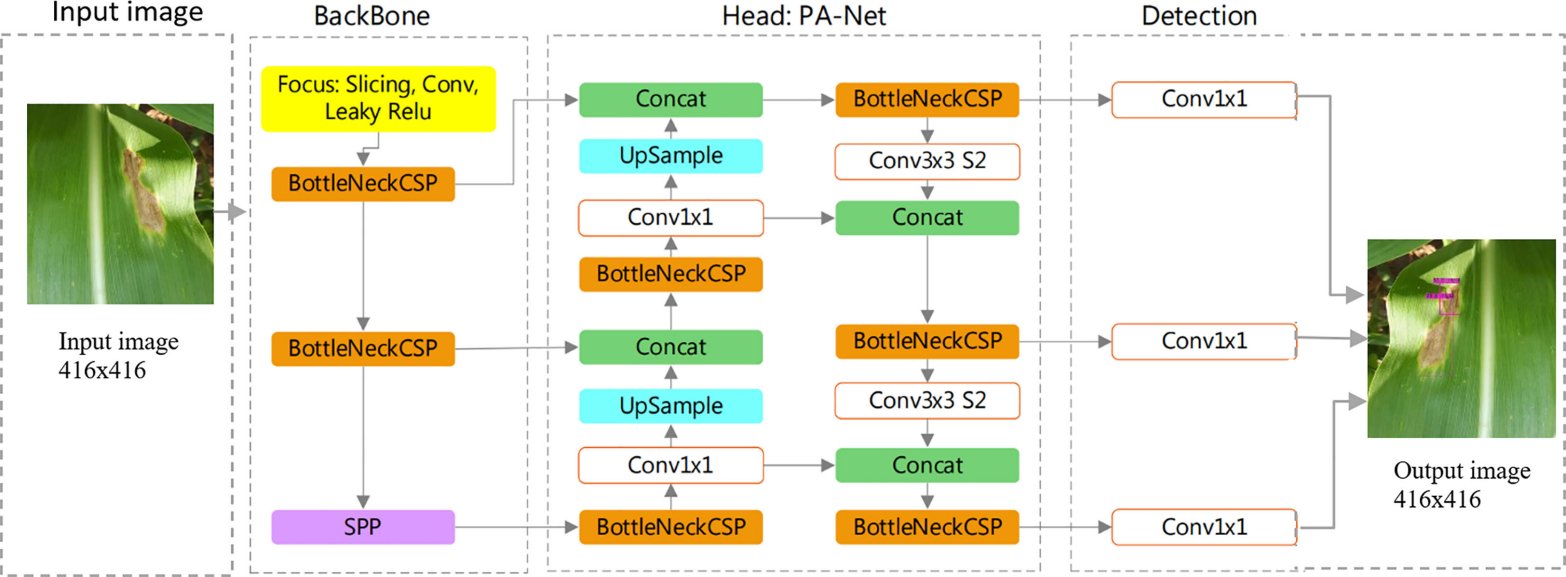
Architecture diagram of YOLOv5s.

The YOLOv5s model is divided into three main components: backbone, head, and detection. The backbone is responsible for collecting the shape image features at different neurons and it is based on CNN. To create image features, the YoloV5s uses the CSP Bottleneck. Layers make up the head, which combines image features to forward them to a prediction process. For feature aggregation, the Yolov5s additionally uses the PAN. The detection procedure includes phases for box and class prediction in addition to using features from the head. The architecture diagram of the YOLOv5s model is shown in **Figure 3** and the hyperparameters used for the training of YOLOv5s model are presented in **Table 1**.

The YOLOv5s has two mappings in the post-processing and the operation process was as described in Equations 3-6.


(3) bx=2× σ(tx)−0.5+cx

(4) by=2× σ(ty)−0.5+cy

(5) $$bw=pw\left(2\times (tw\right){)}^{2}$$

(6) $$bh=ph\left(2\times (th\right){)}^{2}$$

**Figure 4:**
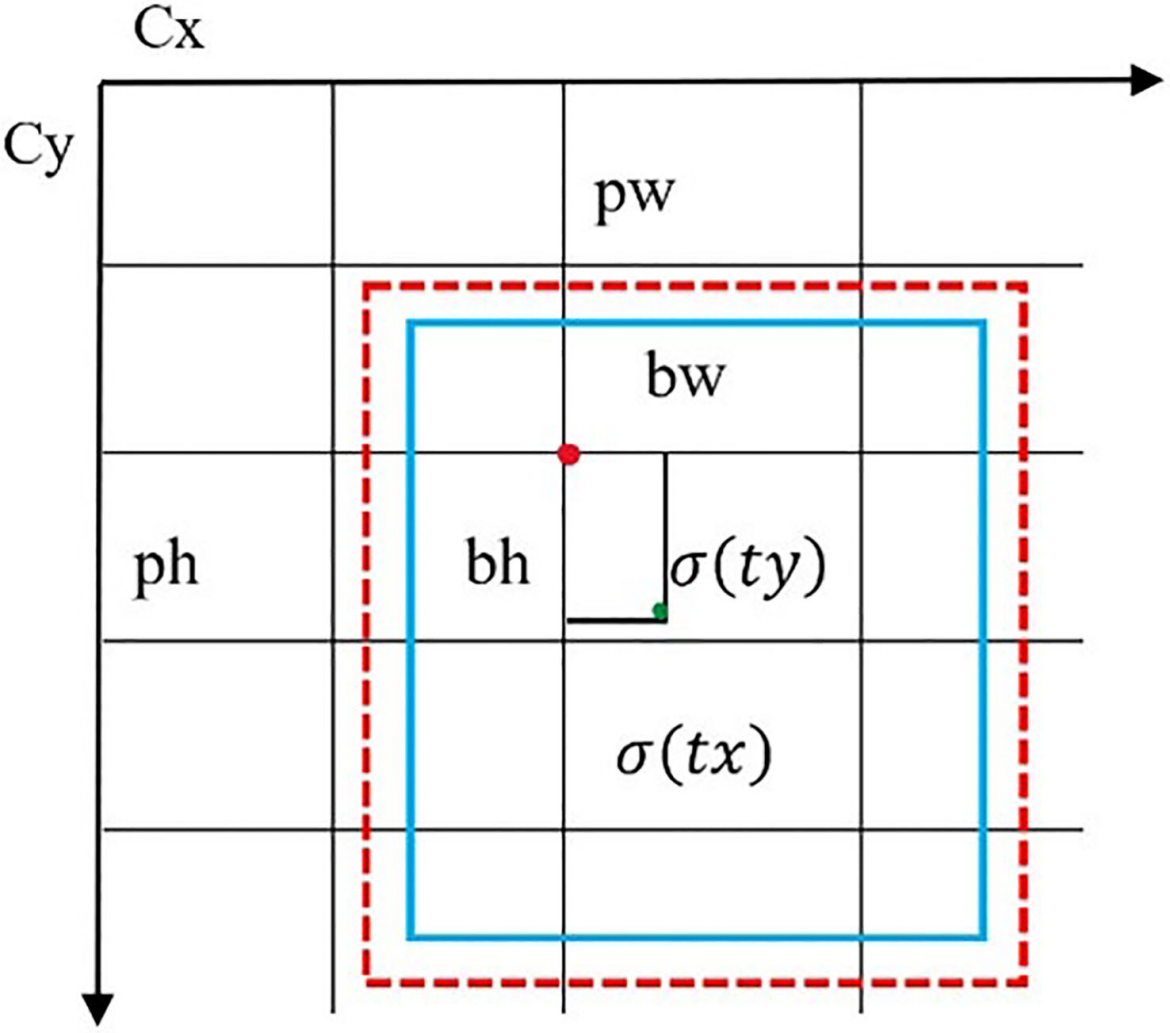
The schematic diagram for calculating the size of the prediction box.

Where “b” is the size of the prediction box, including b_x_, b_y_, b_w_, and b_h_ representing the x-coordinate, y-coordinate, width, and height of the center box, respectively. C_x_ and C_y_ are the side lengths of the cell, p_w_ and p_h_ are the width and height of the prior box. σ  is the sig activation function as shown in **Figure 4**.

### 2.4 YOLOv7s

**Figure 5:**
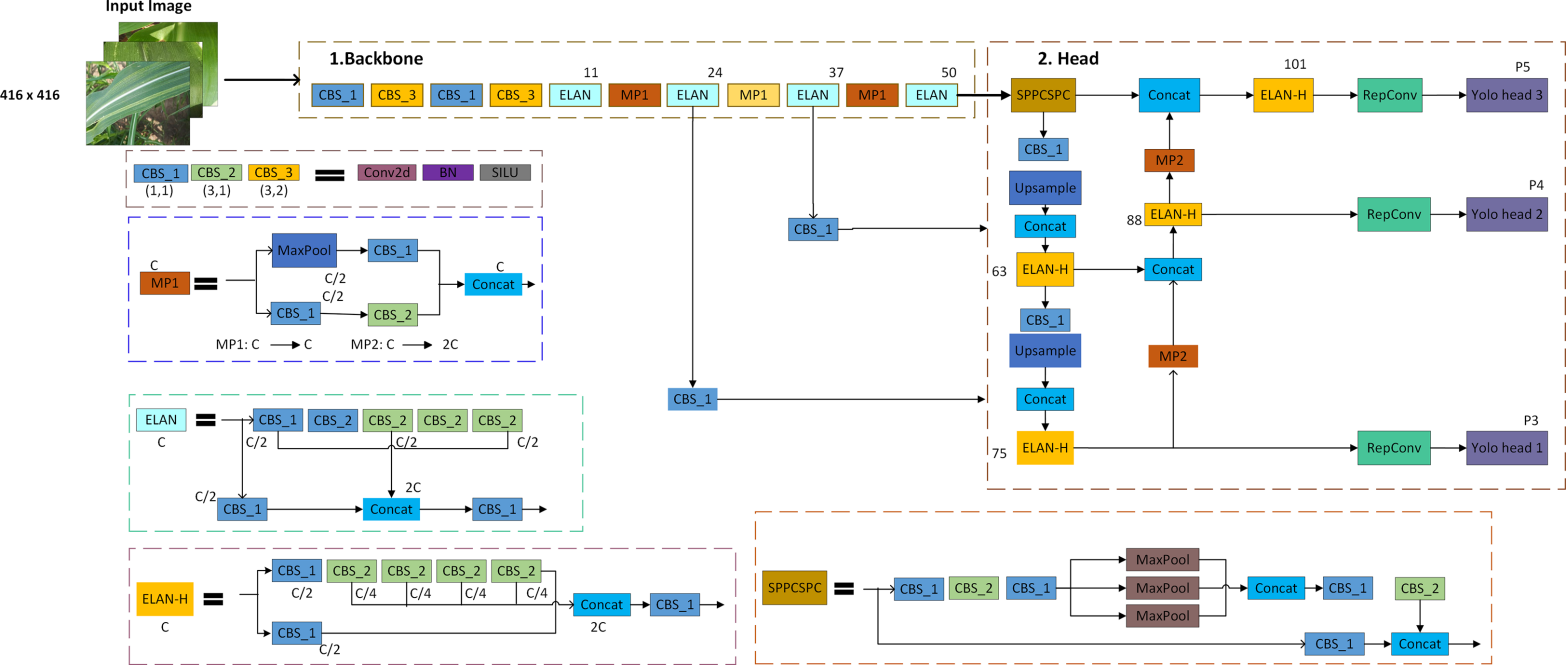
Architecture diagram of the YOLOv7s.

Yolov7s is the smaller version of YOLOv7. It is the advancement of the YOLOv6 in terms of *mAP*, detection speed, and inferencing. The Efficient Layer Aggregation Network (ELAN) is extended in the YOLOv7, which is called the extended ELAN (E-ELAN). Different fundamentals are used to enhance the learning ability of the network named as expand, shuffle, and merge without demolishing the gradient path. It also focused on some methods such as trainable “bag-of-freebies” and optimization modules. Various computational blocks are used to learn more distinct features. After being divided into groups of size “s”, the feature maps from each computational block will be concatenated. The final step will merge cardinality using a shuffled group feature map. The architecture diagram of the YOLOv7 is shown in **Figure 5** and the hyperparameters used to train the YOLOv7 model in this study are reported in **Table 1**.

### 2.5 YOLOv8n

**Figure 6:**
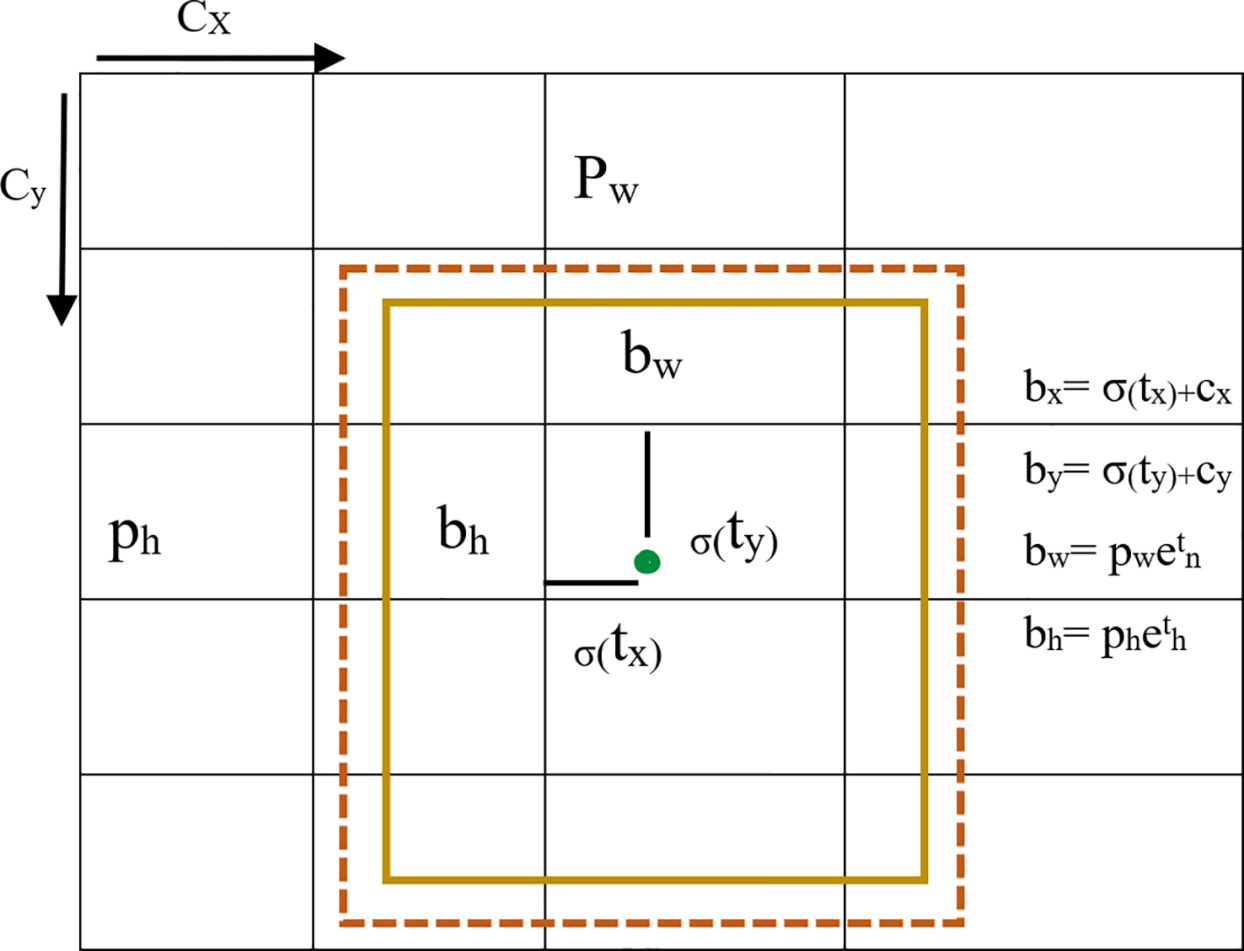
Visualization of an anchor box in YOLOv8n.

The YOLOv8n model is the nano version of the YOLOv8 family because it is small and fast with higher detection results. It can be utilized for object detection and classification in conjunction with instance segmentation and object tracking which makes it SOTA. It was created by Ultralytics, who also developed the YOLOv5 model which is a powerful model. It has several architectural updates and enhancements. It is an anchor-free model which means the model directly predicts the center point of an object in an image rather than the offset from a known anchor box illustrated in **Figure 6**.

**Figure 7:**
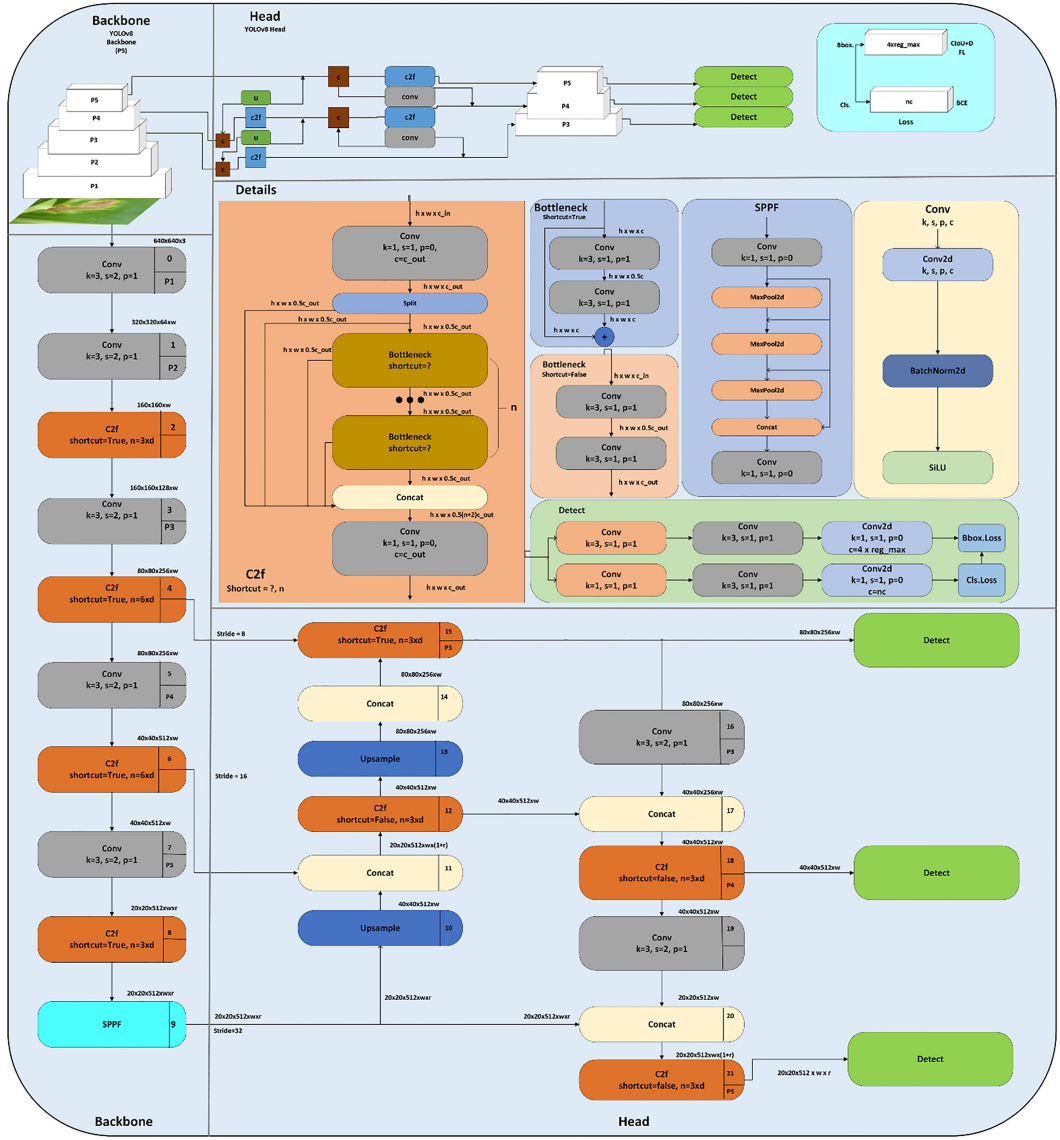
Architecture diagram of YOLOv8n.

The architecture diagram of the YOLOv8 is depicted in **Figure 7**. The SOTA YOLOv8 augments the images at each epoch. The technique used is mosaic augmentation, which stitches the four images and forces the model to learn the new locations. The change in the structure of YOLOv8 is that the c3 module is replaced with the c2f module. In module c2f, outputs from Bottleneck are concatenated, although the output of the last Bottleneck was used in the c3 module. Also, the first 6×6 convolutional layers are replaced with a 3×3 convolutional block in the Backbone module. The main thought behind using the YOLOv8 model was the performance and accuracy boosts during training and inferencing. It is better than the previous versions of YOLO in all aspects (*mAP*, latency, speed, FPS, and size).

## 3 Materials and methods

**Figure 8:**
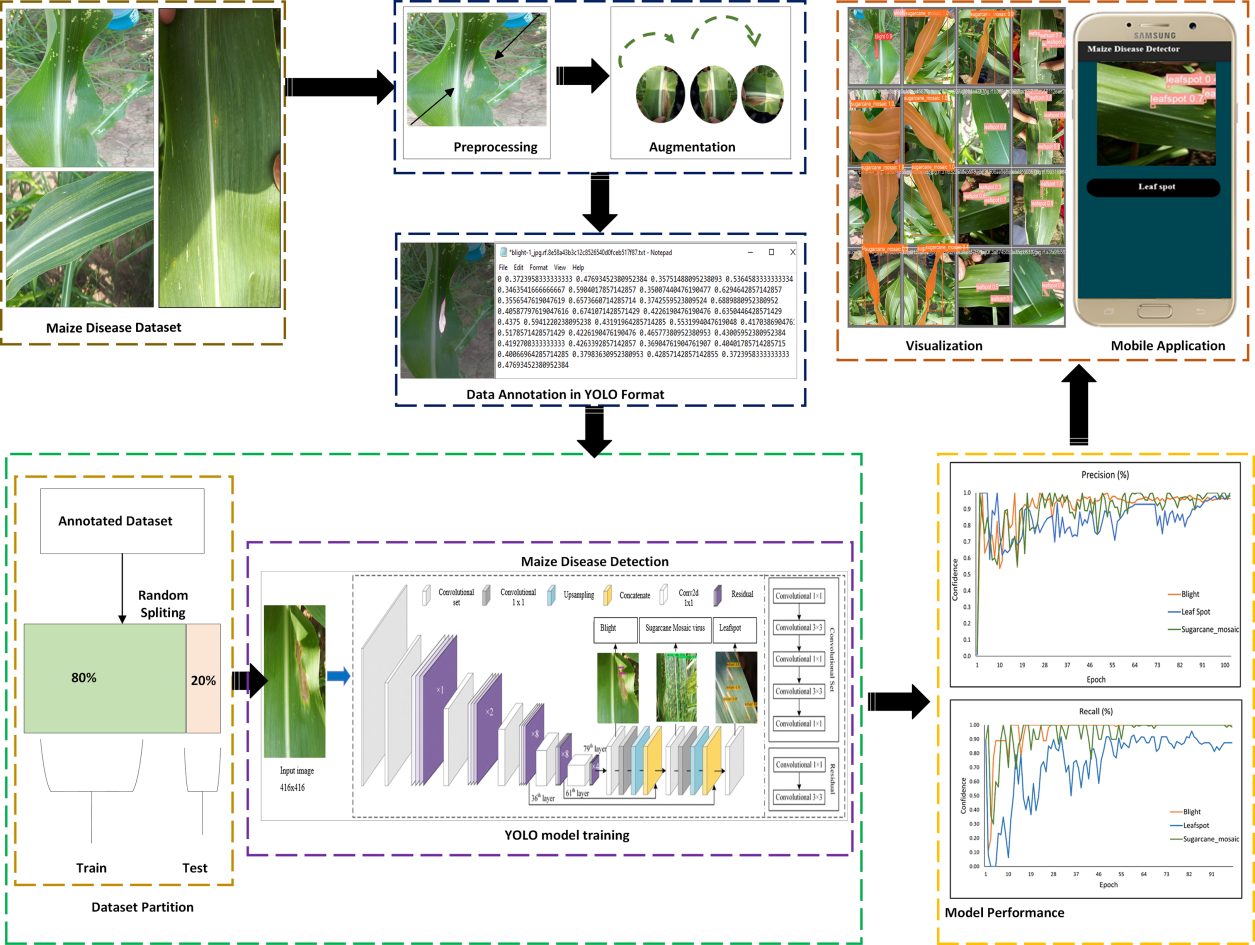
Proposed pipeline of maize disease detection by YOLO detectors.

The quality dataset is the fundamental requirement for building the foundation of the deep learning model because the performance of the deep learning model is highly dependent on the quality, quantity, and relevancy of the dataset. So, the first and crucial step of any deep learning starts with image acquisition. The complete pipeline from image acquisition to model training to smartphone application testing is shown in **Figure 8**. The other activities of the system are listed below and explained in detail in the below sections.

1. The maize crop foliar diseases are collected from University Research Farm Koont (URF), Pir Mehr Ali Shah Arid Agriculture University Rawalpindi (PMAS-AAUR), at different growth stages (initial, middle, and mature), different days of the month with contrasting weather conditions.
2. After data collection, the next step is to perform image preprocessing, including resizing.
3. After that, data augmentation named flipping, rotating, scaling, and cropping was applied.
4. In data annotation, images were annotated/labeled after the experts’ knowledge.
5. The annotated data was then fed into the deep learning model for training.
6. Finally, the aim of our study was achieved by detecting, classifying, segmenting, and tracking of maize crop diseases i.e., blight, leaf spot, and sugarcane mosaic virus in the real-time environment.
7. In the end, results are compared, a suitable model was selected for our problem statement and embedded into the mobile application for real-time detection and tracking.

### 3.1 Dataset collection and description

A detailed description of how we collected the dataset and applied different preprocessing and augmentation techniques are discussed below.

### 3.2 Study area

**Figure 9:**
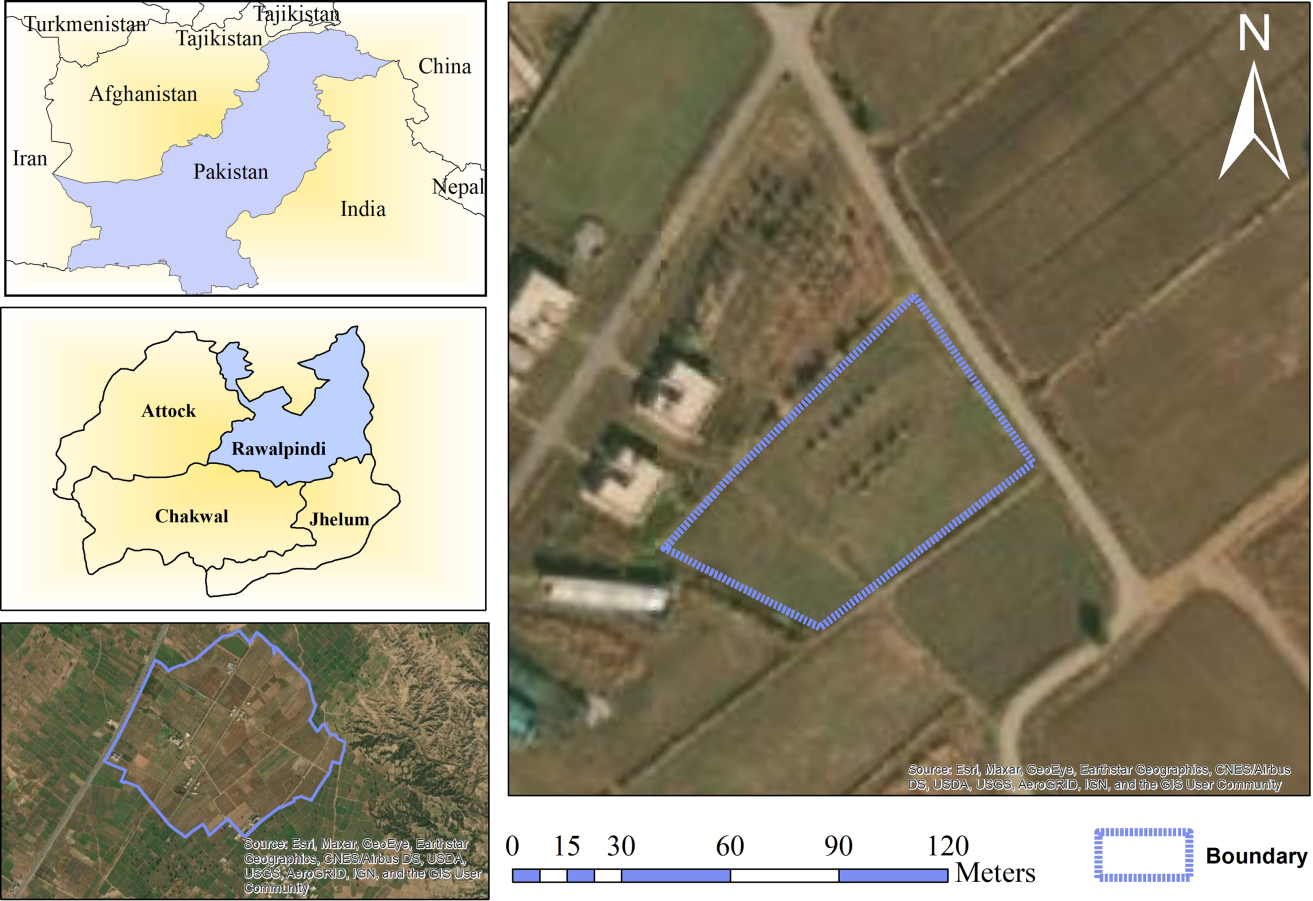
Study Area of Maize Crop.

This study was conducted at URF, Koont PMAS-AAUR, located in Punjab province, Pakistan (33.1166° N, 73.0111° E) as shown in **Figure 9**. The maize variety was Pak Afghoi and planting was done in June. It was sown on about one acre with no application of pesticides and fungicides, the line-to-line distance was 50cm (about 1.64 ft). The seed quantity was 40 kg per acre, and one bag of urea-based fertilizer and one bag of Diammonium Phosphate (DAP) were applied after sowing. The drill sowing technique was used for sowing the seeds.

### 3.3 Experimental setup

All the experiments from training to validation were performed using a Graphics Processing Unit (GPU: Nvidia RTX A4000) on Ubuntu 20.04 LTS operating system. The software and hardware used for model training/testing and their details are presented in **Table 2**.

**Table 2:** Implementation details.

Features	Details
**Software**	Linux 64-bit OS Python 3.8 OpenCV 4.2 CUDA 11.3 cuDNN 8.2
**Hardware**	NVIDIA RTX A4000 RAM 32 GB

### 3.4 Image acquisition

**Figure 10:**
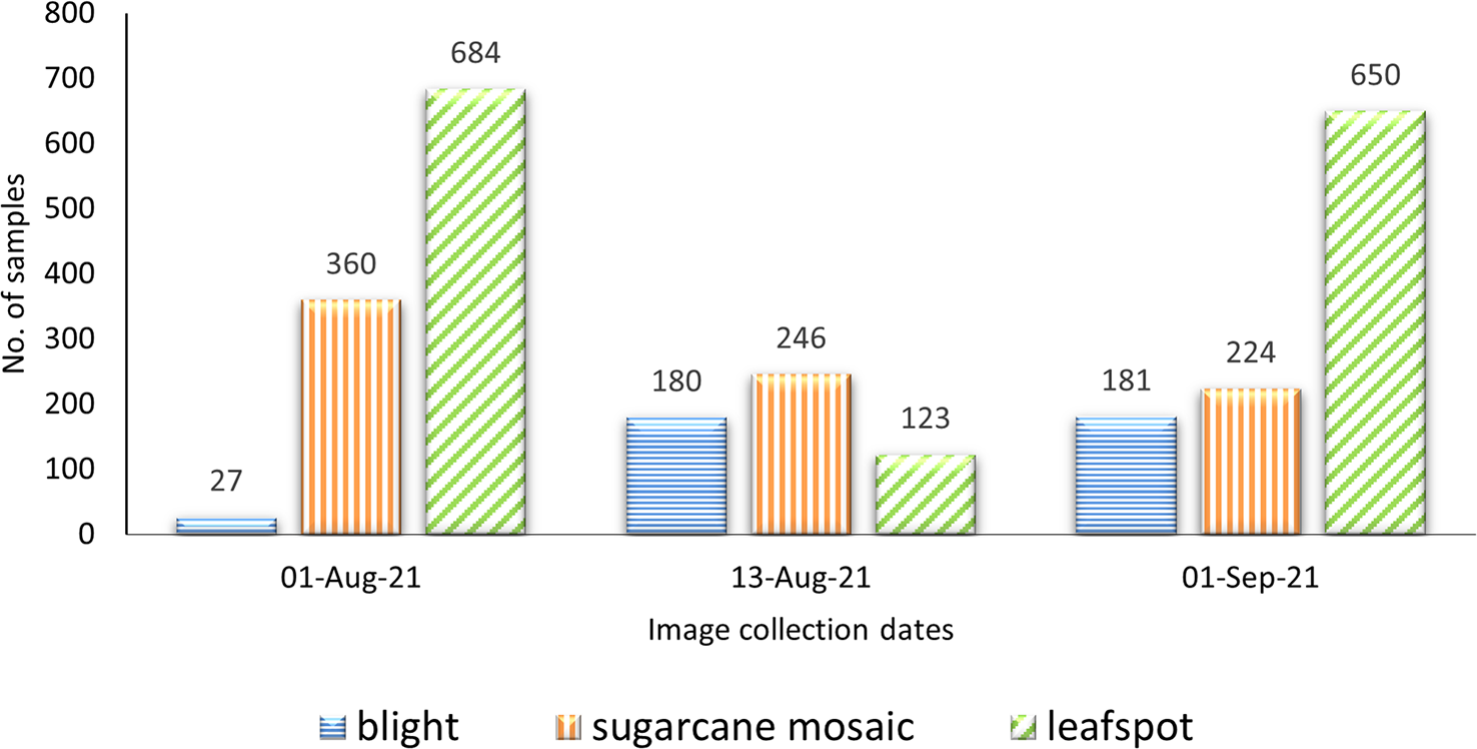
Dataset description.

**Figure 11:**
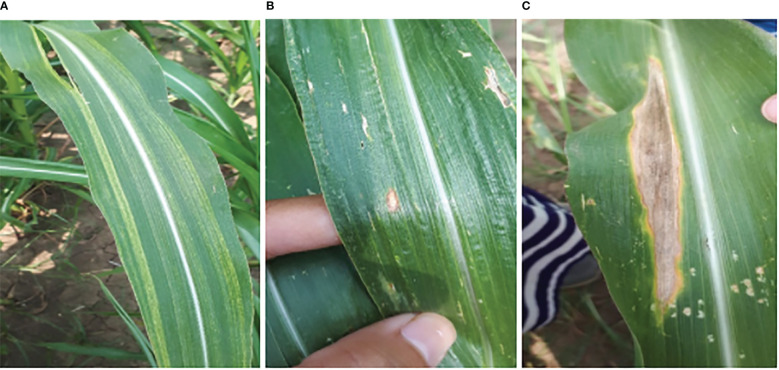
Maize diseases. **(A)** sugarcane_mosaic virus, **(B)** leafspot, **(C)** blight.

At every stage of object recognition research, from the training phase to assessing the effectiveness of recognition algorithms, a suitable dataset is necessary, because the performance of the deep learning models is highly dependent on the input images. To achieve this, 2675 images of maize diseases were collected at various development stages, in contrasting weather conditions, and on different days of the month (June to September) under the naturally diseased environment. The first two months after sowing (June, and July) there were no diseases monitored in the field, so the data was collected after July. The image dataset was captured through the smartphone camera (SAMSUNG Galaxy A7) by maintaining a specific height of 33cm from the leaf surface. The specifications of the smartphone include 16 megapixels (MP) camera, 3GB Random Access Memory (RAM), and a 3.77 MB size of each image. After the collection of images, they were assessed several times by pathologists. The dataset size of maize diseases is illustrated in **Figure 10**, where the x-axis represents the dates when the data was collected, and the y-axis depicts the total number of images. The blue horizontal, orange vertical, and green diagonal lines exhibited blight, sugarcane mosaic virus, and leafspot disease, respectively. The sample images collected from the study area are shown in **Figure 11**.

### 3.5 Image preprocessing

**Figure 12:**
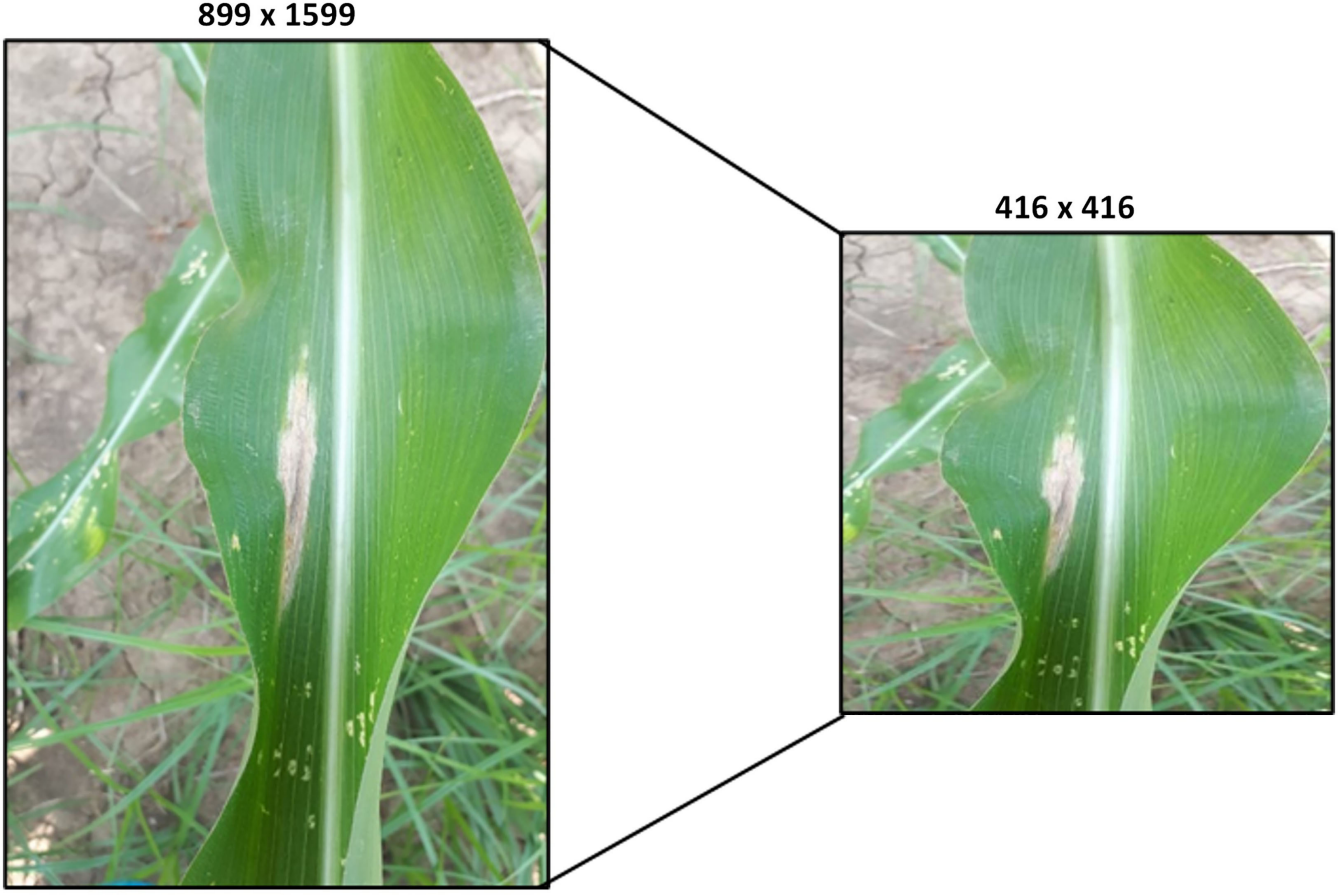
Image resizing.

Image Preprocessing refers to activities performed on images at the most fundamental level (Sarki et al., 2021). Deep learning-based models train faster if the size of the images are smaller. Moreover, the collected raw images vary in size and many architectures of deep learning models require the same image size (Sharma et al., 2020). Also, if there is a difference between the training image and the recognition image, the YOLO shows poor performance (Jeong et al., 2018). Hence, for that purpose, the captured raw images are resized to 416×416 dimensions which is an ideal size for training the YOLO models. When the resizing is applied on an image its pixel values reduced in size and the unwanted region of interest is discarded. This preprocessing method is performed by using a python script with the help of OpenCv library before training the object detection and tracking model (YOLO). **Figure 12** depicted the raw image collected from the real field which is 899×1599 dimensions and the reduced/resized image.

### 3.6 Data augmentation

Programmers can enhance the diversity and the size of the dataset to train the models using different data augmentation techniques (Waheed et al., 2020). It is a known fact that CNN can handle variations in images and classify items even when they are positioned in distinct orientations (Buslaev et al., 2020; Umer et al., 2022). To train CNN, a considerable amount of data is required so that it can discover and retrieve more features. Deep learning models performed best when the size of the dataset is large enough hence for this purpose, we enlarged our dataset by artificially generating samples from our collected dataset to maximize the performance of deep learning models. The most widely used techniques for augmentation were implemented in this research named rotating (70° and 90 °), flipping, scaling, and cropping by using python script.

### 3.7 Image annotation

**Figure 13:**
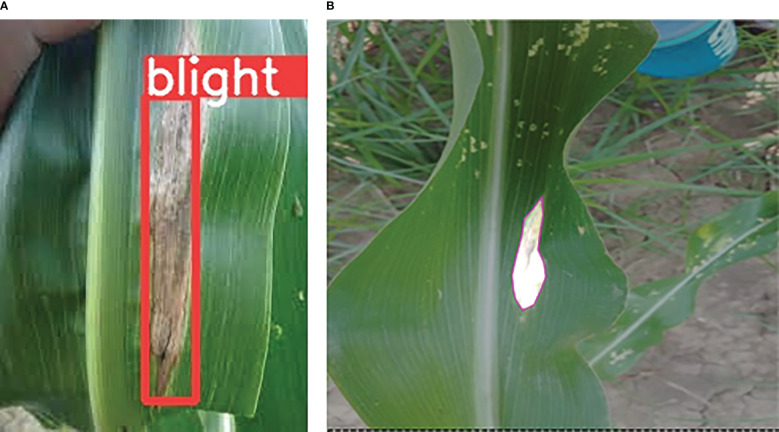
Image annotation for YOLO model training, **(A)** YOLO_mark tool, and **(B)** Roboflow.

Image annotation is the process of labeling the data into different formats such as images, videos, or text files for machines to understand the input data. The annotated/labeled dataset is the most important part of supervised Machine Learning (ML) because models are trained on the input data, machines process that data and produce accurate results. Different annotation tools have been used in the literature such as labelImg, roboflow, yolo_mark, and many others. For YOLOv3, YOLOv4, YOLOv5, and YOLOv7 data annotation, we have used the yolo_mark tool, which is freely available at the AlexeyAB repository. Whereas roboflow which is the computer vision platform that allows users to build computer vision models and image annotations in conjunction with a data augmentation facility was used for YOLOv8n segmentation. The annotated images in both formats, rectangle and segmented are depicted in **Figures 13A**, **B** respectively.

### 3.8 Model performance evaluators

All the models are tested on images that are not part of the training process to check their effectiveness. All the YOLO variants classify the maize diseases very efficiently but YOLOv8n performs best of all. Precision, *mAP*, Loss, and Recall have been used as evaluation indicators in this study for comparative analysis. Gai et al. (2021) have also used these performance evaluation methods for obtaining and comparing the performance of the different models. Precision refers to the model’s ability to recognize only the pertinent objects. Where True Positive (TP) means our model correctly predicts the correct diseased class and False Negative (FN) means the model correctly predicts the incorrect diseased class. The precision is calculated as shown in Equation (7).


(7) $$Precision\;=\;\frac{TP}{TP+FN}$$

Recall (4) refers to the model’s ability to recognize all the pertinent objects. The model can recognize all the detected bounding boxes from the validation set. The false positive (FP) means that the model incorrectly predicts the correct class. The recall is calculated as shown in Equation (8).


(8) $$Recall\;=\frac{TP}{TP+FP}$$

The *mAP* is the mean of the AP of each class. The *mAP* encompasses the trade-off between precision and recall as well as considering both FN and FP and it is calculated as shown in Equation (10), where N is the number of classes.


(9) $$mAP=1/N\sum_{i=1}^{N}AP$$

The loss function of the YOLO model is calculated by Equation (10), where *cls* represents the classification loss, *conf* represents the loss of confidence, and *reg* represents the regression loss. These losses are calculated by the formulas given in Equations 11 to 13. Ji et al. (2021) have used the loss formula for calculating the loss of the YOLO model. The lower the loss value of the model the higher the performance of the model. Where represents the predicted and true probability, $A^{p}\;\text{and}\;A^{\delta }$ exhibits the predicted and true bounding box while the *A_c_*, *C*, and *I* are the desired area, the overlapping area, and the real area respectively.


(10) $$LOSS=\;LOSS_{cls}+\;LOSS_{conf}+\;LOSS_{reg}$$

(11) $$\;LOSS_{cls\;=\;}\sum_{i=0}^{k^{2}}li\;\sum_{c=1}^{c}E\left({\hat{p}}_{i}\left(c\right),\;p_{i}\left(c\right)\right)\;$$

(12) $$LOSS_{conf}=\;\Sigma_{i=0}^{k^{2}}\Sigma_{j=0}^{m}l_{i,j}\;E\left({\hat{c}}_{i},\;C_{i}\right)-\;\lambda_{no}obj\Sigma_{i=0}^{k^{2}}\Sigma_{j=0}^{m}l_{i,j}\;E\left(1.I_{i,j}\right)E\left({\hat{c}}_{i},\;C_{i}\right)$$

(13) $$LOSS_{reg\;=\;}1-IOU\left(A^{p},\;A^{\delta }\right)+\frac{A^{C}-A^{p}-A^{\delta }+I}{A^{C}}$$

### 3.9 Model training and hyperparameters setup

In the original data set, 80%, and 20% of images from each class were selected to form the training set, and validation set, respectively. The image size was set to 416×416 for both training and validation purposes. In the YOLOv8n model proposed in this study, the training process uses the trained weight file of the original YOLOv8n as the initialization parameter. Because different network structures need to be trained in the comparative experiment, and the number of iterations to achieve the optimal detection performance, this study monitors dynamically during training and saves the weight file of the network at every epoch for the selection of the best train model to prevent overfitting. The hyperparameters used for training the YOLOv3-tiny, YOLOv4, YOLOv5s, YOLOv7s, and YOLOv8n are depicted in **Table 1**.

**Figure 14:**
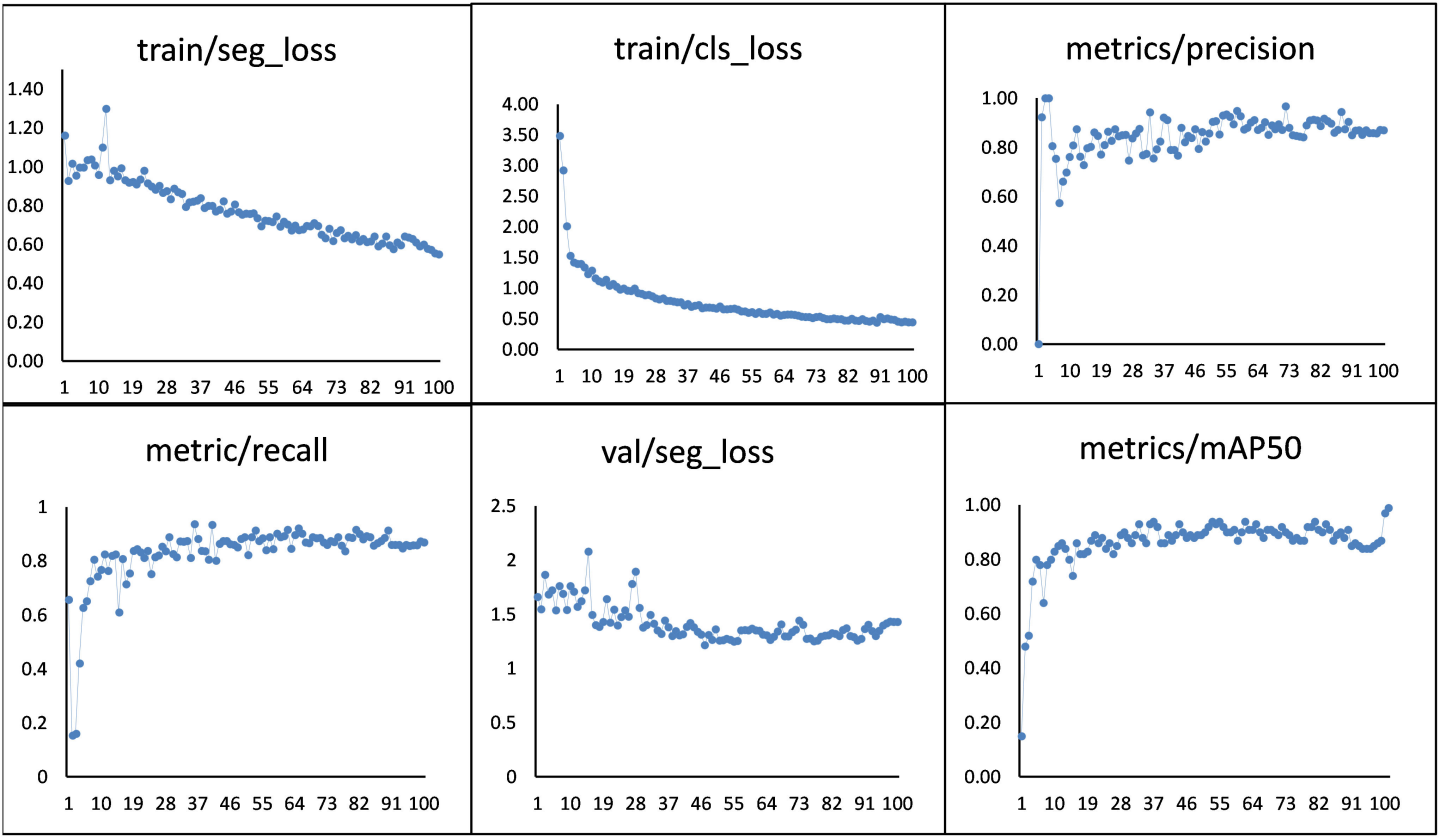
Visualization of model performance evaluators during training of the YOLOv8n model.

The training process was visualized by configuring the weights and biases (Wandb) in this study. It is used to dynamically observe the training status and performance of the model on different iterations. The results of the YOLOv8n model are shown in **Figure 14**. During model iteration, 0-50, the parameters of the model oscillated significantly. When the number of iterations increased, the performance of the model continuously improved. After the 50^th^ iteration, the model index became stable, and the value of Precision reached 0.89 and progressively stabilized. Therefore, the model gets stable and achieved 99.0% *mAP* between 50 to 99 iterations.

The performance of the YOLOv8n predictor for classifying, detecting, segmenting, and tracking the blight, leafspot, and sugarcane mosaic virus diseases in a maize dataset was calculated by the loss function (**Figure 14**). It is used to evaluate the correlation between the given data and the expected outcomes. The lower the loss, the better the performance of the model, and vice versa. There are two types of loss, one represents the training segmentation loss and the other shows the validation segmentation loss of each object. During each epoch, training loss was assessed, and after each epoch testing loss was determined.

## 4 Results and discussion

This section presents the results generated by the applied models, their comparison, and discussion.

### 4.1 Maize disease detection results

**Figure 15:**
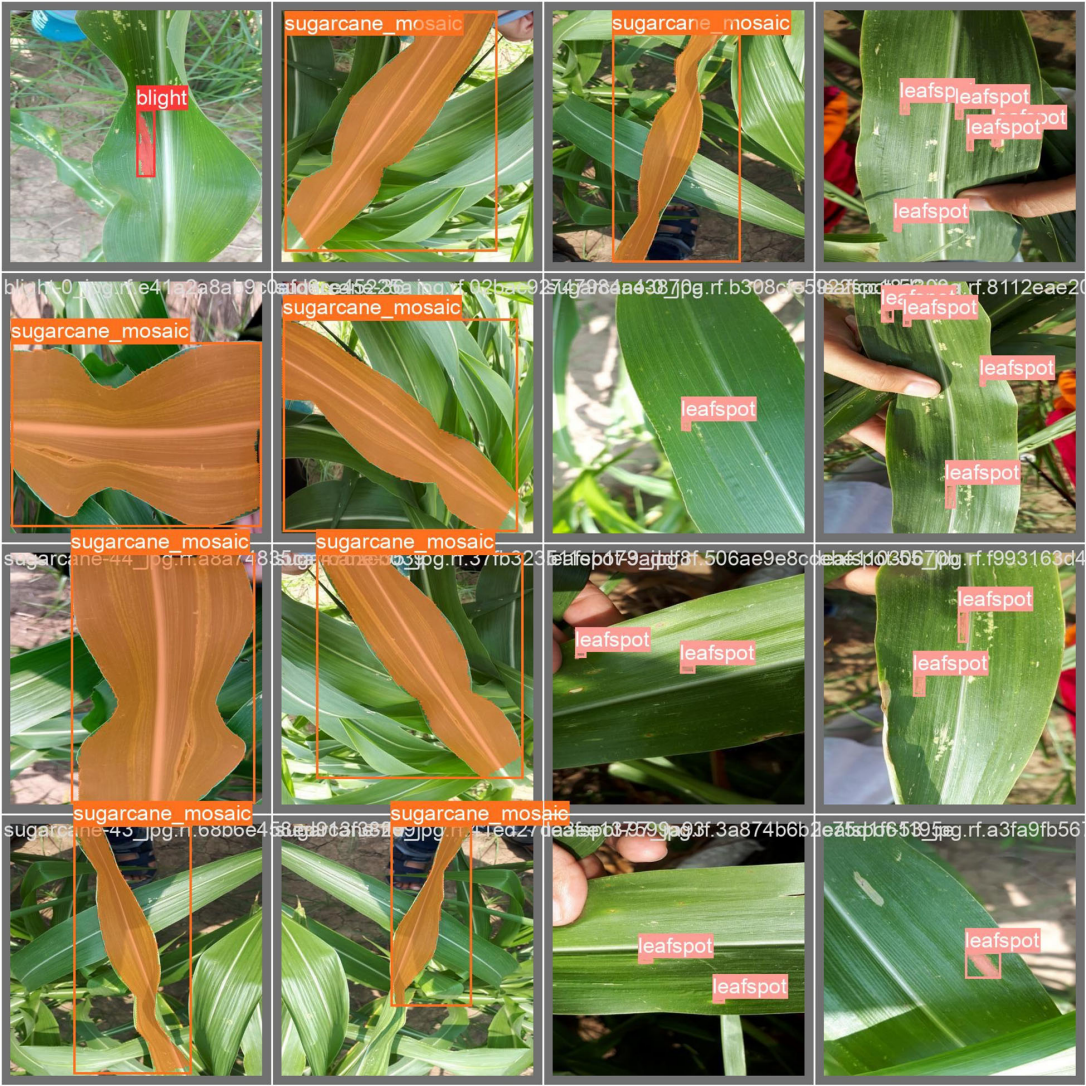
Examples of successful recognition of maize plant diseases using the YOLOv8n model.

The results of the adopted model shown in **Figure 15** were performed to view the detected maize diseases i.e., blight, leaf spot, and sugarcane mosaic virus after training the YOLOv8n model. The unseen images were used for testing to check the feasibility of the model, and the adopted YOLOv8n model predicted the diseases correctly and efficiently with a higher confidence score among other applied models in this study. The threshold value was set to 0.3, which means if the confidence score is greater or equal to 30% then the model categorizes it into the relevant class. It is shown that blight disease is detected by the model with a confidence score of 0.9%, leaf spot disease by 1.0%, and sugarcane mosaic virus disease with 1.0%.

### 4.2 Performance evaluation of detection models

**Figure 16:**
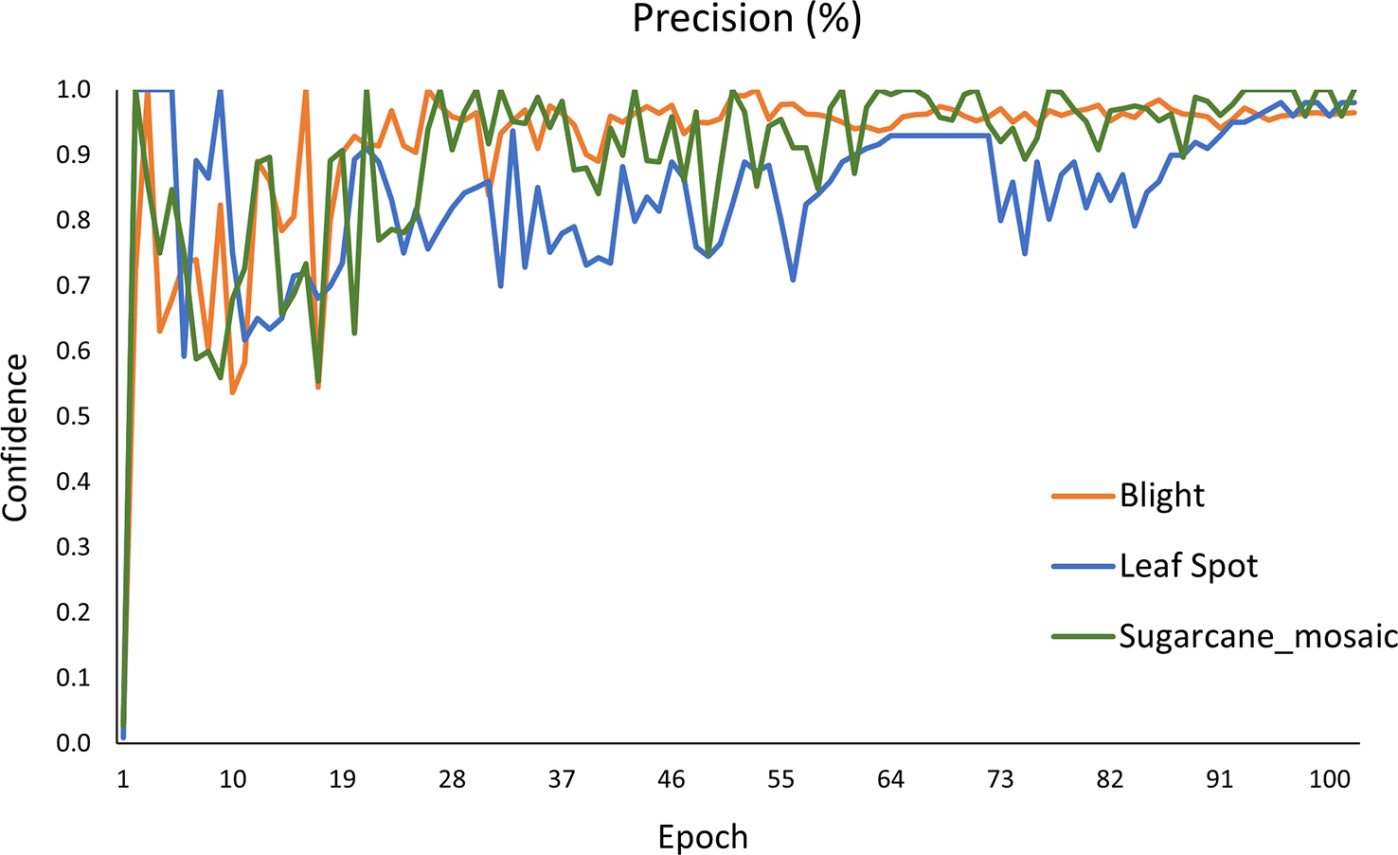
A precision curve of the YOLOv8 model.

**Figure 16** shows the Precision curve of each class. Among all three classes (blight, leafspot, sugarcane_mosaic) of the maize plant diseases, the blight class achieves the highest 0.91% AP, and leafspot gains the lowest 0.84% AP, whereas sugarcane_mosaic obtained the 0.89% AP which is in between blight and leafspot class. Furthermore, it is seen that the precision values of all classes are non significantly different from each other. After the 90^th^ iteration, the model gets stable and achieved above 80% results.

**Figure 17:**
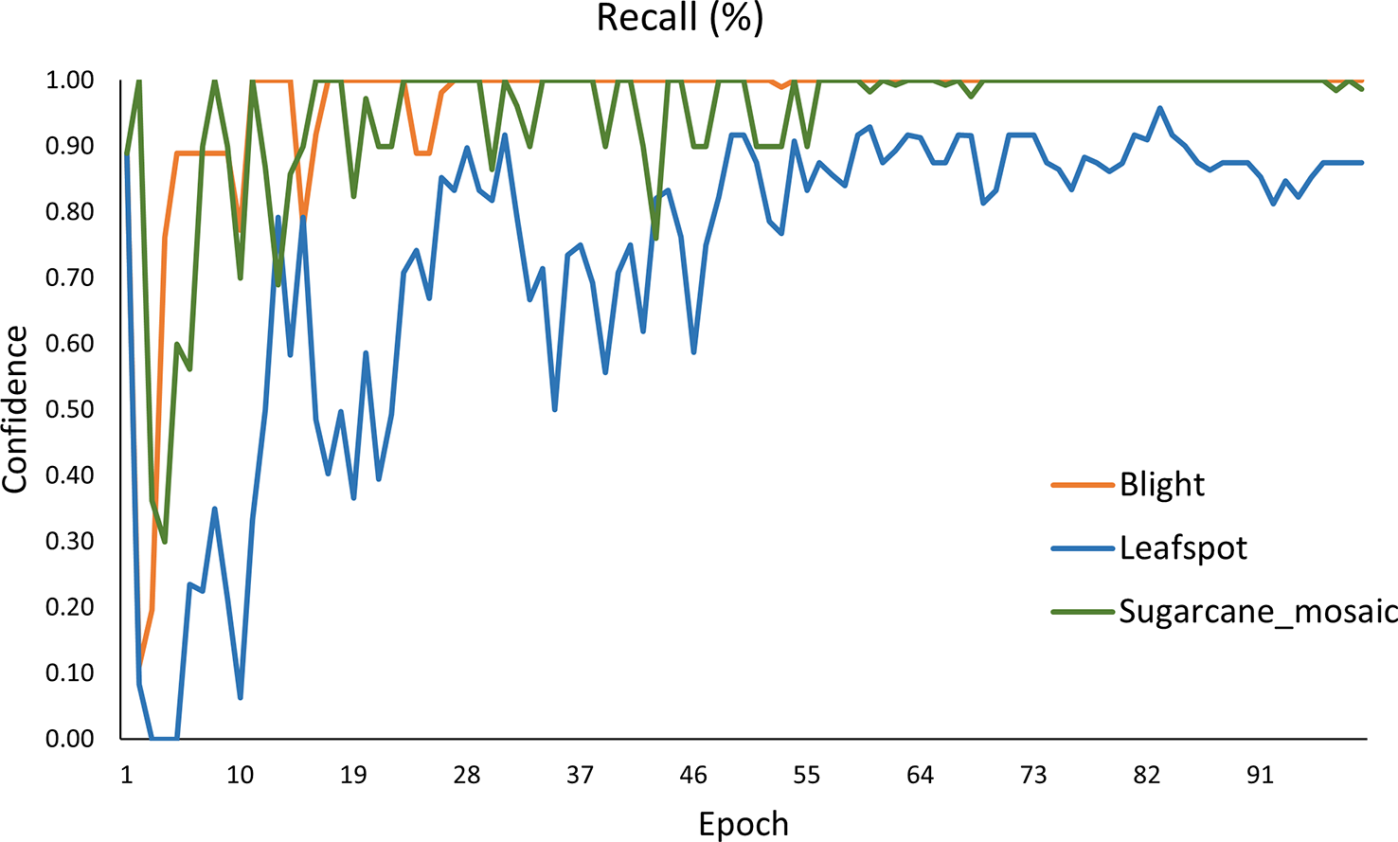
Recall curve of the YOLOv8n model.

**Figure 17** demonstrates the Recall curve of every single class. The value obtained for blight, leafspot, and sugarcane_mosaic is 0.96%, 0.73%, and 0.94% respectively. It is clearly shown that the blight and sugarcane_mosaic get stable after 50 epochs and obtain the highest Recall value, while the performance of the leafspot class fluctuates till 55 epochs and then gets stable, and achieves satisfactory results.

### 4.3 Mobile application

**Figure 18:**
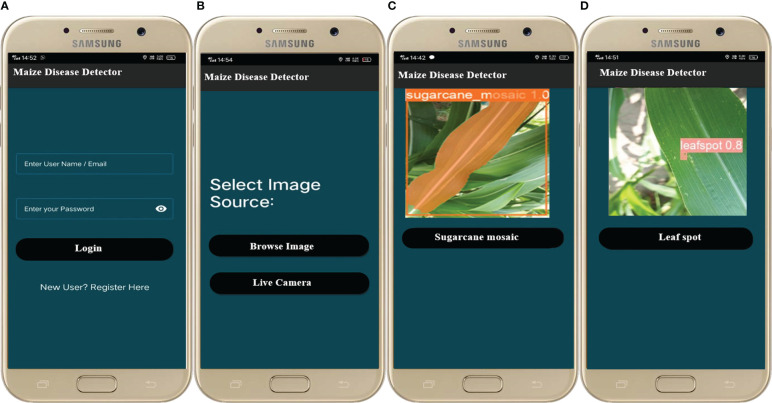
Screenshots of the maize disease detection application **(A)** login/register **(B)** Image selection **(C)** detected leaf spot disease **(D)** detected sugarcane mosaic virus disease.

**Figure 19:**
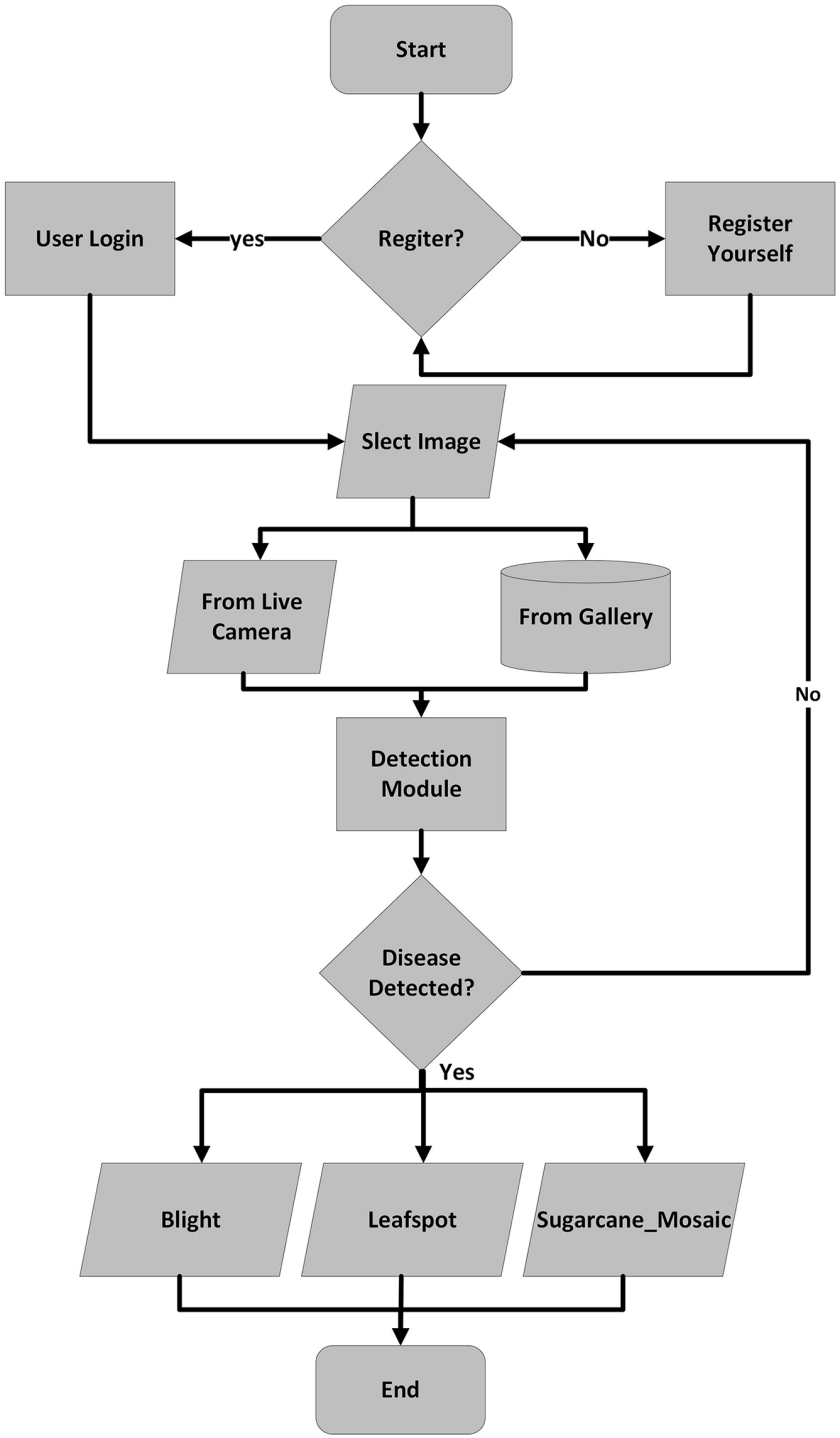
Flowchart of the smartphone application.

The best-trained model was embedded into a mobile application for real-time detection, segmentation, and tracking which helps the user to timely detect the diseases of the maize crop efficiently. First, the model’s result file is converted to .tflite using the TensorFlow lite converter library^1^ and deployed into the mobile application. The mobile app was designed using java language^2^ on Android Studio using the Android Java Development Kit^3^ (JDK). Finally, the diseases are detected/recognized by the TensorFlow lite interpreter library^4^ The interface of the mobile application is illustrated in **Figure 18** and the full working of the application is depicted in **Figure 19**. There are different modules/activities in the mobile application. The first activity obtained the user login detail just for keeping the record of the user for next time. For this purpose, a user must select a valid username and a valid email address as well for account verification. Based on the provided information, the user account will be created, and this information could be updated anytime in the future. For real-time disease detection of maize crop, the user can either select the image from the mobile already saved in the mobile gallery, or he/she could capture the new image in real-time from the field for disease detection. Once the image is selected or captured, then it is forwarded to the detection model. After that, the next activity is opened which will show the detected disease of the maize crop.

### 4.4 Comparative analysis between applied models

The experimental results of the YOLOv3-tiny, YOLOv4, YOLOv5s, YOLOv7s, and YOLOv8n are presented in **Table 3** where 0, 1, and 2 represent the blight, leafspot, and sugarcane mosaic virus disease class respectively. It is reported that the YOLOv5s achieved the best results on blight (class 0) with 99.50% AP, YOLOv8n performed best on leafspot (class 1) disease, and sugarcane_mosaic disease (class 2) with AP of 99.01% and 99.07% respectively. It is investigated that the YOLOv8n obtained better results than other applied versions of YOLO in terms of accuracy, segmentation, and tracking. Moreover, this study also investigates the other performance evaluators including precision, recall, and loss. It is reported in the table that the precision value of the YOLOv7s model achieves higher and the loss value it achieves is smaller than other YOLO variants. The higher recall score was gained by the YOLOv4.

**Table 3:** A comparative analysis between YOLO versions used in this study.

Yolo Variants	Ap			*mAP* (%)	Precision (%)	Recall (%)	Loss (%)
	0	1	2				
**YOLOv3-tiny**	78.11	72.43	57.71	69.40	0.81	0.43	1.09
**YOLOv4**	98.71	97.11	96.57	97.50	0.89	0.98	2.13
**YOLOv5s**	99.40	91.70	73.60	88.23	0.95	0.89	0.01
**YOLOv7s**	98.70	92.20	89.00	93.30	1.0	0.95	0.005
**YOLOv8n**	99.05	99.01	99.07	99.04	0.88	87.66	0.78

**Figure 20:**
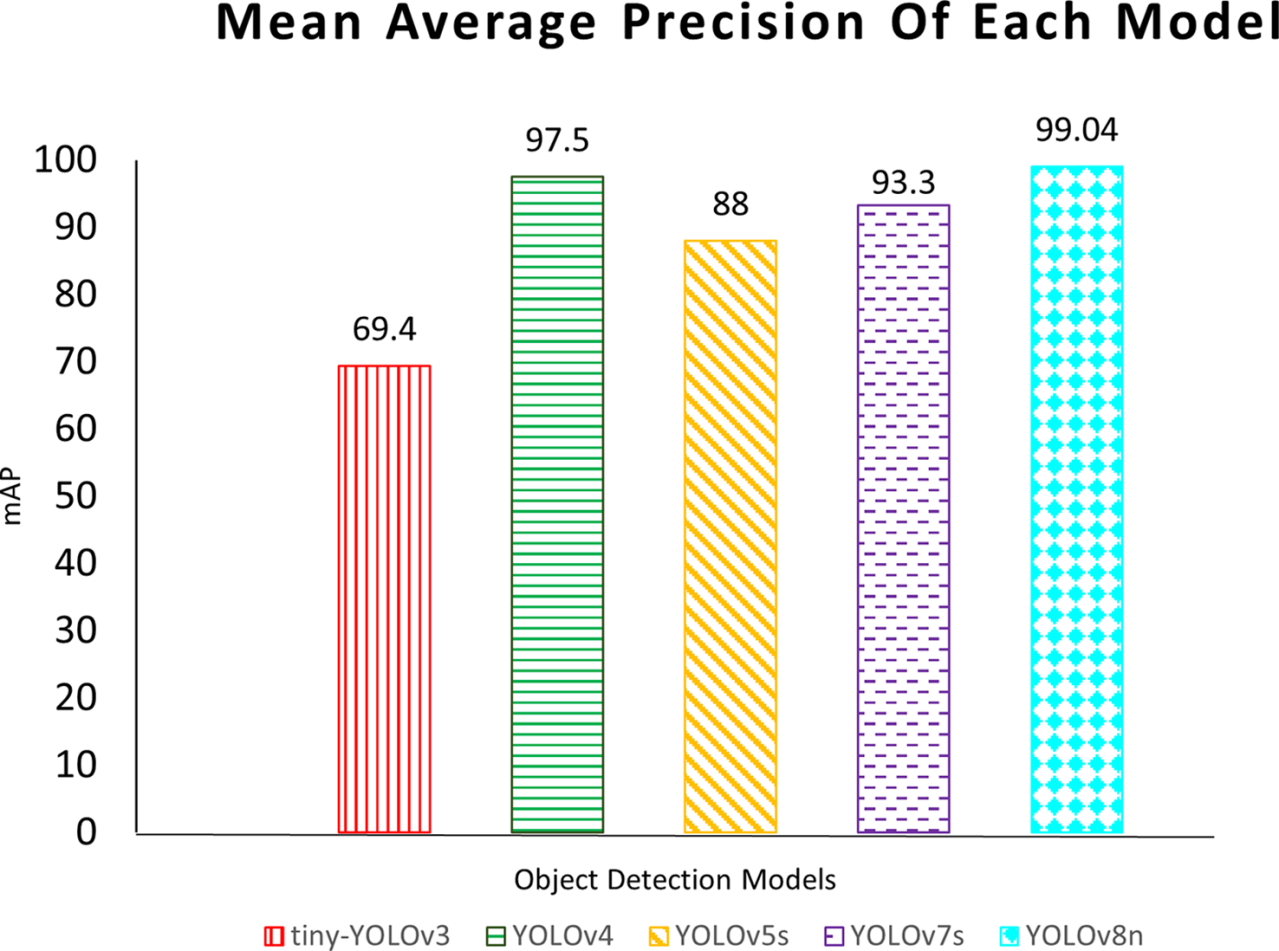
Comparison between applied models in this study.

The results (*mAP*) of each model applied in this study are depicted in **Figure 20**, the red color vertical lines, green color horizontal lines, yellow color diagonal lines, purple color horizontal strips, and aqua color diamond grids show the YOLOv3-tiny, YOLOv4, YOLOv5, YOLOv7s, and YOLOv8n respectively. The comparative results illustrate that the YOLOv8n outperforms all.

The results of the comparative analysis of deep learning-based disease detection algorithms of our study with the other studies are expounded in **Table 4**. The authors (Austria et al., 2022; Li et al., 2022b) have used the YOLOv5 algorithm for the detection of apple and corn respectively, and they have achieved 90% and 97% results. Li et al. (2022b) used YOLOv5s, which is the smaller version of the YOLO model, and they obtained 93.10% accuracy. Liu and Wang (2021) have worked on the disease detection of the tomato plant and obtained 92.39% *mAP*. In this study, we have applied YOLOv3, YOLOv4, YOLOv5, YOLOv7s, and YOLOv8n for the detection, segmentation, and tracking of maize plant diseases in the real-time environment and achieved 69.40%, 97.50%, 88.23%, 93.30%, and 99.04% results respectively.

**Table 4:** Comparative analysis of real-time detection systems.

State-of-the-art	Plant	Algorithm	Accuracy (%)
(Liu and Wang, 2020)	Tomato	YOLOv3	92.39
(Li et al., 2022b)	Vegetables	YOLOv5s	93.10
(Li et al., 2022b)	Apple	YOLOv5	90.00
(Austria et al., 2022)	Corn	YOLOv5	97.00
Our study	Maize	YOLOv3-tiny, YOLOv4, YOLOV5s, YOLOv7s, YOLOv8n	69.40%, 97.50%, 88.23%, 93.30%, 99.04%

## 5 Conclusion

Plant diseases have long been a major issue in agriculture. Early disease detection through deep learning models can overcome the spread of diseases at an early stage and minimize the losses. In this work, well known deep learning-based object detection model YOLO is used for the detection of diseases in maize crop. Five different versions of YOLO including YOLOv3-tiny, YOLOv4, YOLOv5s, YOLOv7s, and YOLOv8n are used and the dataset for this purpose is collected from a real environment where crop was grown in a university research farm Koont. Data collected in this work contains three different diseases including blight, leaf spot, and sugarcane mosaic virus, and is pre-processed using different data augmentation techniques. Models trained for disease detection were able to accurately detect, classify, segment, and track the diseases with a high confidence score. A comparison between different versions of YOLO models confirms that the YOLOv8n model achieves the best detection results among all and meets the requirements of real-time detection. The *mAP* value achieved by this model was 99.04%. To increase the system’s usability the best-trained model with higher detection accuracy was embedded into the mobile application for real-time disease detection and classification. In the future, these kinds of models could be integrated with the Unmanned Ariel Vehicles (UAV) for real-time crop monitoring and management.

## Data availability statement

The raw data supporting the conclusions of this article will be made available by the authors, without undue reservation.

## Author contributions

All authors listed have made a substantial, direct, and intellectual contribution to the work, and approved it for publication.

## Footnotes

1
https://www.tensorflow.org/lite/models/convert

2
https://www.oracle.com/java/

3
https://docs.oracle.com/javase/8/docs/

4
https://www.tensorflow.org/lite/guide/inference
